# Cryptogenic stroke and atrial fibrillation in a real-world population: the role of insertable cardiac monitors

**DOI:** 10.1038/s41598-020-60180-6

**Published:** 2020-02-24

**Authors:** Maria Vittoria De Angelis, Vincenzo Di Stefano, Raffaella Franciotti, Nanda Furia, Enrico Di Girolamo, Marco Onofrj, Massimiliano Faustino

**Affiliations:** 1 Department of Neurology, “SS Annunziata” Hospital, Chieti, Italy; 2 Department of Neuroscience, Imaging and Clinical Sciences, “G. d’Annunzio” University, Chieti, Italy; 3 Department of Cardiology and Cardiac Surgery, “G. d’Annunzio” University, Chieti, Italy

**Keywords:** Neuro-vascular interactions, Neurophysiology

## Abstract

The incidence of atrial fibrillation (AF) in cryptogenic stroke (CS) patients has been studied in carefully controlled clinical trials, but real-world data are limited. We investigated the incidence of AF in clinical practice among CS patients with an insertable cardiac monitor (ICM) placed for AF detection. Patients with CS admitted to our Stroke Unit were included in the study; they received an ICM and were monitored for up to 3 years for AF detection. All detected AF episodes of at least 120 sec were considered. From March 2016 to March 2019, 58 patients (mean age 68.1 ± 9.3 years, 67% male) received an ICM to detect AF after a CS. No patients were lost to follow-up. AF was detected in 24 patients (41%, AF group mean age 70.8 ± 9.4 years, 62% male) after a mean time of 6 months from ICM (ranging from 2 days to 2 years) and 8 months after CS (ranging from 1 month to 2 years). In these AF patients, anticoagulant treatment was prescribed and nobody had a further stroke. In conclusion, AF episodes were detected via continuous monitoring with ICMs in 41% of implanted CS patients. AF in CS patients is asymptomatic and difficult to diagnose by strategies based on intermittent short-term recordings. Therefore, we suggest that ICMs should be part of daily practice in the evaluation of CS patients.

## Introduction

In 20–40% of ischemic strokes, a definitive cause is not identified, despite extensive evaluation^1^; this condition has been defined as “cryptogenic stroke” (CS)^1–6^. Many hypotheses have arisen to find an explanation for the stroke occurrence, but there is no consensus^7^. Atrial fibrillation (AF) is a well-known cause of ischemic stroke and about 15% of strokes are attributable to a documented AF^8,9^. Moreover, AF is the most common cause of cardioembolism in patients older than 70^10,11^. Unfortunately, AF can evade conventional monitoring strategies of patients with acute cerebral ischemia, thus supporting its possible role in CS^12^. The diagnosis of AF has clinical relevance, as randomized clinical trials have shown that anticoagulation reduces the risk of stroke in patients with AF^13,14^. However, it is required to document AF to initiate anticoagulant therapy after ischemic stroke and, in the absence of documented AF, antiplatelet agents are recommended^15^. A diagnosis of AF can influence the management of CS, because the presence of AF changes the pharmacological therapy to anticoagulant prescription. Indeed, it has been shown that anticoagulation is superior than antiaggregation in preventing further strokes in patients with AF^16^. The risk of stroke in patients with AF can be estimated by the CHA_2_DS_2_-VASc score^17^, but, even in the presence of strong suspicion, AF may not be detected in the acute phase of ischemic stroke, due to its paroxysmal and asymptomatic nature^18–21^. For this reason, many strategies have been explored to improve detection of AF, ranging from in-hospital monitoring, serial electrocardiography and Holter monitoring and the use of external events (or loop recorders) or insertable cardiac monitors (ICM)^22–24^.

Recently, it has been demonstrated that ICMs improve the diagnostic sensibility to detect many arrhythmias, especially AF in patients with CS, with detection rates (DR) to 25%^20,22,24^. Many reports and single-centre studies have appeared, but there was a lack of randomized trials until 2014^25–28^. The CRYSTAL-AF study was the first randomized study demonstrating the superiority of ICMs to standard care monitoring in CS^24,29,30^. However, these trials reflected results in patients with specific conditions, inclusion and exclusion criteria. More recent studies explored the incidence of AF in real-world cohorts of patients with an ICM implanted after a CS (cardiac event monitor) with a relatively brief follow-up^26,31^. Only a few reports have explored the long-term DR of AF in the CS population^32^.

In the present real-world study, we show the incidence of AF detected by ICM in a cohort of 58 CS consecutively recruited patients with 3 years of follow-up. These data reinforce literature data on long-term monitoring (up to 2 years) with the use of ICMs in CS patients. The wide use of ICMs in clinical practice could significantly enhance the detection of AF and improve the prognosis, allowing early initiation of anticoagulant therapy, in a relevant percentage of patients with CS.

## Methods

The study was performed according to the declaration of Helsinki and its later amendments, and it was approved by the ethical committee of the “SS Annunziata” hospital of Chieti. All patients included in the study consented to the use of their data for research purposes and signed informant consent for study participation.

### Patient population

We included all patients admitted to the Neurological Clinic of “SS Annunziata” hospital of Chieti, which received an ICM after a cryptogenic stroke. A stroke was classified as “cryptogenic” if extensive testing failed to reveal a clear aetiology. The workup included: complete neurological examination and CHA_2_DS_2_-VASc score, 24-hours ECG monitoring, transthoracic echocardiography, screening for thrombophilic states (under 55 years of age), transcranial and neck Doppler ultrasound, magnetic resonance angiography, computed tomography angiography of the head and neck. Each patient was monitored for at least 4 months after device insertion. Then, patients were divided into two groups based on the presence (AF group) or absence (no-AF group) of AF (which was recorded by ICM after stroke).

### ICM device

The ICM device (Reveal LINQ, Medtronic) is a small cardiac monitor that is inserted into the subcutaneous tissue over the heart. This device detects and records automatically AF, irrespective of heart rate or symptoms^32^. ICM recordings were monitored constantly to detect possible ECG anomalies during the study. The Medtronic CareLink^TM^ Network was used to transmit the device data remotely. The device will automatically initiate wireless transmission every night for episodes recorded during the prior day. Patients may also initiate a full transmission of all data contained within the device’s memory at any time.

Data were collected in the One Hospital ClinicalService project (Clinical Trial Registration Information: http://clinicaltrials.gov/ct2/show/NCT01007474), an observational medical care project that periodically provides our site with information and analysis of clinical and device data regarding our stroke patient population wearing a Medtronic ICM. Main aim of the project is to help healthcare providers in improving patient outcomes and understanding of their patients’ care or patients’ management. The project was approved by Hospital Medical Director and conformed to the Declaration of Helsinki. All patients provided their informed consent for data collection and analysis.

Follow-up visits were scheduled at 1 month and every 3 months thereafter until study closure, with unscheduled visits in the event of symptom occurrence or after the transmission of ICM data, if advised by the investigator.

AF was defined as an episode of irregular heart rhythm, without detectable P waves, lasting more than 120 seconds. An expert cardiologist (FM) validated all recorded AF episodes. Only confirmed AF episodes were included in the analysis.

### Statistical analysis

We reported continuous variables as mean with standard deviation and categorical variables as numbers (percentage). We compared categorical variables among AF and no-AF groups with the Chi-square test and continuous variables with the Mann-Whitney test. We performed all tests using SPSS (v22) and established the level of significance at the 0.05 level.

### Ethical standards

the study was performed in accordance with the ethical standards laid down in the 1964 Declaration of Helsinki and its later amendments, and it was approved by the local ethical committee of Chieti. All patients gave their informed consent prior to their inclusion in the study.

## Results

### Patients

Table 1 summarizes demographic and follow-up data from our patients. From March 2016 to March 2019, 58 patients (mean age 68.1 ± 9.3 years, 67% male) were consecutively enrolled and received an ICM to detect AF after a CS. Among all 58 patients, the most frequent risk factors of stroke were hypertension (67% of patients), hypercholesterolemia (62%), diabetes (26%), smoking (16%) and previous stroke (14%, Table 1). No patients had heart failure. The Chi-square test showed that hypertension, diabetes, smoking, hypercholesterolemia, coronary artery disease, baseline and discharge NIHSS were not significantly different between the AF and no-AF group; the only exceptions were previous stroke (26% in the AF and 6% in the no-AF group, p = 0.025), and CHA_2_DS_2_-VASc score (4.9 ± 1.2 in the AF and 4.1 ± 1.0 in the no-AF group, p = 0.035).

**Table 1:** Demographic data, CHAD_2_VASC_2_ score, risk factors, stroke severity, echocardiography measures and temporal monitoring for AF in all patients and in AF and no-AF group.

	Total (n = 58)	AF group (n = 24)	No-AF group (n = 34)	P value
Age, years	68.1 ± 9.3	70.8 ± 9.4	66.2 ± 8.6	0.06
Male, n (%)	39 (67%)	15 (62%)	24 (71%)	0.91
Time to ICM (from CS), days	83 ± 74	68 ± 59	94 ± 86	0.38
Time to AF (from CS, days)	NA	257.8 ± 182.9	NA	/
Time of follow-up (from ICM, days)	906 ± 288	938 ± 268	885 ± 301	0.68
Time of follow-up (from CS, days)	989 ± 270	1006 ± 250	977 ± 285	0.14
CHA2DS2-VASc score	4.4 ± 1.4	4.9 ± 1.2	4.1 ± 1.0	0.035*
Hypertension	39 (67%)	17 (70%)	22 (65%)	0.90
Diabetes	15 (26%)	5 (22%)	10 (29%)	0.051
Smoking	9 (16%)	3 (13%)	6 (17%)	0.81
Hypercholesterolemia	36 (62%)	13 (54%)	23 (68%)	0.94
Heart failure	0	0	0	/
Coronary artery disease	7 (12%)	4 (17%)	3 (9%)	0.74
Previous stroke	8 (14%)	6 (26%)	2 (6%)	0.025*
Baseline NIHSS score	4.40 ± 2.14	3.79 ± 1.50	4.84 ± 2.56	0.63
Discharge NIHSS score	1.77 ± 1.60	1.83 ± 1.23	1.72 ± 1.82	0.15
Echocardiography: EF, %	61.55 ± 3.96	59.3 ± 5.0	62.9 ± 3.0	0.18
Echocardiography: left atrial volume index (LAVI), ml/m^2^	33.45 ± 9.94	38.7 ± 9.1	30.1 ± 8.0	0.012*

Data are shown as means ± standard deviation for continuous variables or numbers (percentage) for categorical variables. AF, atrial fibrillation; ICM, insertable cardiac monitor; CS, cryptogenic stroke; NA not applicable; EF, ejection fraction. *p < 0.05.

### ICM implantation and follow-up

ICM was implanted after a mean time of 83 ± 74 days (ranging from 3 to 453 days) from CS (no significant difference among groups, Table 1). No patients were lost to follow-up. All patients were followed-up for a mean of 906 ± 288 days after device insertion (no significant difference among groups). Patients of the no-AF group were monitored for a mean time of 24 months after CS ranging from 6 months to 3.5 years, and a mean time of 21 months after ICM implantation ranging from 3 months to 3 years. In the no-AF group the follow-up time from ICM implantation was lower than 6 months in 7 patients (18%), 12 months in 10 patients (26%), 18 months in 5 patients (13%) and 24 months in 8 patients (21%). Eight patients (21%) of the no-AF group were followed for a time greater than 2 years. All patients tolerated ICM and none required it to be removed.

### AF detection

AF was detected in 24 patients (41%, 62% male), which received the ICM after a mean time of 68 days (ranging from 6 to 317 days) from CS. Mean age was 70.8 ± 9.4 years for AF group and 66.2 ± 8.6 years for no-AF group (71% male). No-AF group was younger (p = 0.06) than AF group. The no-AF group received the ICM after a mean time of 94 ± 86 days from CS. The AF and no-AF group were not significantly different for the elapsed time between the CS and the ICM implantation (p = 0.38).

**Figure 1:**
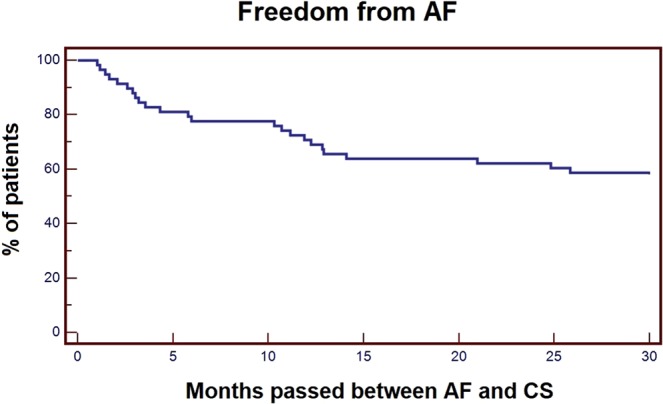
Kaplan-Meier cumulative curve for all patients when the time passed between the occurrence of AF and the CS is used as the survival time variable. AF, atrial fibrillation; CS, cryptogenic stroke.

AF was detected after a mean time of 6 months from ICM (ranging from 2 days to 2 years) and 8 months after CS (ranging from 1 month to 2 years). Specifically, AF was detected within 6 months after CS in 13 patients (54% of AF group, 22.4% among all CS), between 6–12 months after CS in 5 patients (20.8% in AF group, 8.6% among all CS), between 13–18 months after CS in 3 patients (12.5% in AF group, 5.2% among all CS). Only 3 patients (12.5% in the AF group, 5.2% among all CS) showed AF after 21, 25 and 26 months after CS respectively. Figure 1 shows the Kaplan-Meier cumulative curve for AF when the time passed between the occurrence of AF and the CS is used as the survival time variable.

### AF characteristics and burden

AF was asymptomatic in all patients in the AF group. Twenty-four patients had single or multiple episodes of AF; ventricular arrhythmia and atrioventricular blocks were reported in one and two patients respectively.

In the AF group we registered a mean overall AF burden of 32 ± 52 days and a mean daily AF burden of 57 ± 82 minutes for patient. The maximum duration of AF episode was 681 ± 635 minutes (range from 2 minutes to 4 days). We excluded a patient from analysis because he developed persistent AF.

### Echocardiography

All patients were evaluated through transthoracic echocardiography; there was no significant difference among ejection fraction among groups (Table 1), but echocardiography demonstrated higher left atrial volume index (LAVI) in the AF (38.7 ± 9.1 ml/m^2^) compared to the no-AF group (30.1 ± 8.0 ml/m^2^, p = 0.0119).

### Anticoagulant treatment and outcome

In all AF patients, anticoagulant treatment was prescribed and nobody had another stroke. However, in the no-AF group, one patient (3% of the no-AF group, 1.7% among all CS) had further strokes after six months from the first CS.

## Discussion

The incidence of AF in CS patients has been studied in carefully controlled clinical trials, but real-world data are limited. A significant number of patients with CS can suffer from AF which is not detected during the standard diagnostic procedures. Indeed, AF may often be paroxysmal and asymptomatic; many patients with AF have long period of sinus rhythm, making short duration non-continuous monitoring suboptimal for excluding AF^18,33^.

There are several non-invasive strategies, in fact, for detection of AF after stroke^34^, including in-hospital monitoring^35^, serial electrocardiography^36^, Holter monitoring, the use of external events (or loop recorders)^22,23^ and long-term outpatient monitoring^37^. Studies that evaluated holter monitoring ranging from 24 to 72 hours, have a low yield for AF detection after stroke (3–5%)^38^.

The association between AF and cryptogenic stroke was strengthened by intensive monitoring using ICM^22–24^. Continuous electrocardiographic monitoring with ICM has been shown to be superior to conventional follow-up in detecting AF in the population with cryptogenic stroke^24,30^.

Further studies demonstrated that long-term continuous electrocardiographic monitoring with ICMs is significantly more effective than intermittent monitoring strategies (24-hour, 48-hour, and 7-day Holters and monthly 24-hour Holters) for identifying AF in patients with previous cryptogenic stroke^25,39^.

Current guidelines suggest to consider implanted loop recorder in stroke to optimize detection of silent AF, but the use of prolonged monitoring after CS is currently left to physician discretion^14,40^.

Even though in the last decades, randomized and controlled studies had demonstrated the importance of cardiac monitoring via ICMs devices in cryptogenic stroke patients^24,29,30^, there are only a few real-life studies with follow-up longer than 12 months. In a recent study by Ziegler *et al*.^32^ AF was diagnosed in 21.5% of CS patients by 24-months follow-up. Our study represents the first real-life cohort of CS patients in Europe with a follow-up longer more than 24 months.

Previous studies reported that the percentages of AF were 16% at 30 days and 21% at 2 years in a population of 280^41^ and 1247^32^ patients with a recent CS, respectively. At 24 months follow-up, we found a significantly higher DR of AF (41%) in comparison with previous literature (χ^2^ = 6.4 p = 0.01 and χ^2^ = 4.3 p = 0.04)^32,41^. This result has to be interpreted in light of several considerations. We suppose that CHA_2_DS_2_-VASc score, which estimates the risk of stroke in patients with AF^17^, may contribute to explain such a high overall DR in our cohort. In accord with previous studies, CHA_2_DS_2_-VASc score was higher in the AF than in the no-AF group (p = 0.035), confirming that thromboembolic risk correlates with AF risk. Moreover, the higher frequency of previous strokes in the AF group can be correlated to the embolic etiology predisposing to recurrence. Then, previous stroke can be considered as a marker of higher risk for AF in patients with CS.

We hypothesize, indeed, that the age can influence the higher value of CHA_2_DS_2_-VASc score in the AF group. Our data showed that AF group was older than no-AF group (p = 0.06), confirming the role of age on AF detection^42,43^. In addition, patients from our cohort (68.1 ± 9.3 years) are older than patients from previous literature (i.e. Ziegler’s cohort, DR 21.5% at 24 months, mean age 65.3 ± 13 years; Sanna’s cohort, DR 8.9% at six-month, mean age 61.6 ± 11.4 years, etc)^24,32^; similarly, in Gladstone’s cohort the DR and mean age were higher respect to the Sanna’s cohort (DR 16.1% at three months, mean age 72.5 ± 8.5 years)^24,41^. These considerations, taken together with our data, support the hypothesis that the occurrence of AF in CS patients was influenced by age. Unfortunately, previous studies used different methods for AF detection: in the study of Gladstone^41^ AF was defined when it was longer than 30 seconds while other authors defined AF episodes lasting at least 2 minutes^32^. This issue may partially explain differences in the DR.

Interestingly, in our study LAVI was higher in the AF then in the no-AF group. LAVI has been reported to be associated with the risk of AF and then it might be useful for identifying individuals with a high risk of AF and thromboembolic complications, improving treatment outcomes^44,45^. Atrial disease or myopathy forms the substrate for AF and underlies the potential for atrial thrombus formation and subsequent stroke.

Also, in our study at 6 months follow-up DR was higher (22%) than in a previous one (8.9%)^24^. Even if we hypothesize that the age is the major contributor to the difference in DR among studies, it cannot be excluded that the shorter time interval between stroke and ICM placement in our cohort might have contributed to a higher DR. We suppose that a considerable percentage of arrhythmic events occurs in the early stages of stroke and may be lost by a delay in positioning the device. Finally, a selection bias could have reduced DR scores in previous studies that included patients with transient ischemic attacks^24,41^. Many diseases can mimic transient ischemic attacks^46^, thus generating a possible enrolment of patients who may not have vascular disease.

In the present real-world study, we show a high incidence of AF detected by ICM in a cohort of 58 CS patients consecutively recruited and followed for 3 years. Interestingly, our data support the idea that ICM can detect AF in CS patients after a complete diagnostic workup. Also, we provide evidence of the efficacy of anticoagulation in the AF group, as none had a recurrence of stroke, at the difference with one patient in the no-AF group.

Moreover, AF was detected within 6 months after CS in 54% of AF group and within 12 months in further 13% (overall 70% at 12 months) with a growing DR in the first 24 months from ICM positioning (Fig. 1).

Finally, we found that patients in the AF group had higher frequency of previous strokes and higher CHA_2_DS_2_-VASc scores, as well as atrial volumes detected by transthoracic echocardiography. These data confirm that patients with a more severe cardiovascular profile are more prone to develop AF and arise the possibility of a connection between “atrial disease” and AF. However, more data are needed to deepen these relations.

A last consideration should be made about the prescription of anticoagulants in silent AF, as nowadays it is widely discussed. In our cohort, all patients had silent AF with single or multiple episodes of AF with a high AF burden, and all patients had high risk according to European Guidelines, allowing a full indication for oral anticoagulants^47^; moreover, we recorded lower stroke recurrence rates in the AF respect to the No-AF group (0 versus 3% after 30 months of follow-up), supporting the prescription of anticoagulants.

### Limitations

Some technical issues should be pointed out: the device used in the study requires at least 2 minutes of AF to be detected and therefore episodes less than 2 minutes are not detected. On the contrary, many artifacts can mimic AF with a possibility of false positives; however, the confirmation of the diagnosis through an expert cardiologist can contain this possibility.

The most important limitation of this study is the sample size. Indeed, even if implantation of ICM was proposed to all patients with CS arriving in our stroke unit, some patients refused the implantation due to the invasiveness of the procedure. It would be interesting to reproduce our result in a larger cohort of CS patients.

This is an open label prospective study without comparison with other forms of monitoring; future studies should compare ICMs with other types of continuous or intermittent monitoring. Another limitation emerges from the time interval between the stroke and the device implantation that appears to be in an apparent delay in the no-AF group respect to the AF group, even if this delay was not statistically significant; this might have reduced the DR in the AF group, representing a factor that leads to underestimation of DR.

Further studies are needed to establish the role of ICM in CS in real-world populations.

## Conclusions

This study describes an AF DR of 41% after 30 months in patients with a previous negative standard intermittent monitoring, thus reinforcing literature data on long-term monitoring of CS patients with the use of ICMs. Moreover, the study shows that CS patients with previous strokes, high CHA_2_DS_2_-VASc scores and high atrial volumes are more likely to have or develop AF during the follow-up; this subgroup of CS patients are ideal candidate for ICM implantation.
